# Direct optical activation of human IRE1 identifies unique patterns of transcriptional and post-transcriptional mRNA regulation in the unfolded protein response

**DOI:** 10.1016/j.jbc.2025.111067

**Published:** 2025-12-18

**Authors:** Jacob W. Smith, Damien B. Wilburn, Vladislav Belyy

**Affiliations:** 1 The Ohio State Biochemistry Program, The Ohio State University, Columbus, Ohio, USA; 2 Center for RNA Biology, The Ohio State University, Columbus, Ohio, USA; 3 Department of Chemistry and Biochemistry, The Ohio State University, Columbus, Ohio, USA

**Keywords:** IRE1, UPR, Endoplasmic Reticulum, ER stress, optogenetics, splicing, transcriptomics, nanopore sequencing, NMD, RIDD

## Abstract

Inositol-requiring enzyme 1 (IRE1) is one of the three known sensor proteins that respond to homeostatic perturbations in the metazoan endoplasmic reticulum. The three sensors collectively initiate an intertwined signaling network called the unfolded protein response (UPR). Although IRE1 plays pivotal roles in human health and development, understanding its specific contributions to the UPR remains a challenge due to signaling crosstalk from the other two stress sensors. To overcome this problem, we engineered a light-activatable version of IRE1 and probed the transcriptomic effects of IRE1 activity in isolation from the other branches of the UPR. We demonstrate that 1) oligomerization alone is sufficient to activate IRE1 in human cells, 2) IRE1’s transcriptional response evolves substantially under prolonged activation, and 3) the UPR induces major changes in mRNA splice isoform abundance in an IRE1-independent manner. Our data reveal previously unknown targets of IRE1’s transcriptional regulation and direct degradation. Additionally, the tools developed here will be broadly applicable for precise dissection of the UPR in diverse cell types, tissues, and organisms.

The endoplasmic reticulum (ER) performs critical roles in eukaryotic cell biology, including protein maturation and lipid metabolism. The dynamic balance between cellular demands and ER capacity is regulated by an intricate signaling network called the unfolded protein response (UPR) (1, 2). The UPR in metazoans dynamically combines the outputs of the three known ER-resident sensor proteins: protein kinase R-like ER kinase (PERK), activating transcription factor 6 (ATF6), and inositol requiring enzyme 1 (IRE1) to direct the cell towards homeostasis, adaptation, or programmed cell death (3). The UPR plays a major part both in human development and in diseases such as type 2 diabetes, neurodegeneration, and several types of cancer including multiple myeloma and triple-negative breast cancer (4, 5, 6, 7, 8, 9, 10). Therefore, developing a robust model of UPR signaling is necessary to understand cellular proteostasis, to develop therapeutics against ER-associated diseases (11), and to learn how to prevent drug-induced ER stress (12, 13). However, building such a model has been complicated by the interconnected nature of the UPR and the experimental difficulties associated with isolating the individual contributions of the three sensor proteins.

IRE1, the most evolutionarily conserved of the three UPR sensors and the focus of this study, is a transmembrane protein composed of a stress-sensing domain within the ER lumen, a single-pass transmembrane domain, and a bifunctional kinase/RNase domain in the cytosol (14, 15, 16, 17). In its inactive state, IRE1α (the primary paralog in mammals (18); simply IRE1 hereafter) is thought to exist as unphosphorylated monomers or dimers (19) that further oligomerize in response to ER stress (20, 21). This oligomerization enables *trans*-autophosphorylation of the kinase domain (22) and subsequent activation of the RNase domain (23). Fully activated IRE1 recognizes and cleaves the unspliced mRNA of the transcription factor X-box binding protein 1 (*XBP1u*) at two locations (24, 25) that are then ligated together by the cytosolic RTCB complex (26, 27) into a form that encodes the “spliced” XBP1 protein (*XBP1s*) (28). While XBP1u protein is not thought to be an active transcription factor, XBP1s upregulates hundreds of genes including those encoding critical components of protein-processing machinery in the ER (29, 30, 31). IRE1 has also been reported to cleave and induce degradation of other mRNA substrates in a process called regulated IRE1-dependent decay (RIDD) (32, 33), which may be impacted by IRE1’s oligomeric and phosphorylation state (34). RIDD is thought to be a mechanism by which IRE1 can independently augment the slow transcriptional response of XBP1s and rapidly reduce expression of a select group of genes. In addition, IRE1 has been reported to cleave a larger pool of RNA targets lacking the endomotif associated with RIDD targets in a process termed RIDDLE (RIDD lacking endomotif) (34).

Substantial effort has been invested in understanding IRE1’s specific contributions to the mammalian UPR. Published experimental strategies include induced expression of XBP1s (35), UPR activation with suppression of the IRE1 pathway (36, 37, 38), UPR activation with suppression of the other UPR sensors (39), and activation of the IRE1–XBP1 axis with small molecules (40, 41, 42). These foundational studies have revealed major conserved effects of IRE1 activation, though every approach comes with caveats: *XBP1u* is not the only substrate of IRE1 (32, 33, 34), so controlling XBP1s expression does not capture the entirety of IRE1 function; acute ER stress may obscure certain effects of IRE1 signaling that occur under the mild levels of ER stress that cells are more likely to encounter *in vivo*; and, finally, pharmacological activation of IRE1 may induce off-target effects (42) and does not directly change the oligomeric state of IRE1, which may play a role in its substrate preferences (34). As a result, despite decades of study, the full scope of effects and regulation of IRE1 signaling is still unclear.

Furthermore, critical aspects of IRE1 regulation remain uninvestigated, such as the temporal dynamics of the IRE1 transcriptional program. The effects of chronic IRE1 activation, as seen in type II diabetes, are easily obscured during laboratory activation of acute ER stress, as IRE1 is known to be attenuated by prolonged PERK activity (43, 44, 45). IRE1 is also involved in functions beyond ER proteostasis, including cell differentiation (46, 47, 48, 49) and regulating lipid imbalances (50), but the aforementioned experimental challenges have prevented an in-depth characterization of these additional roles. Current research also illustrates the importance of aspects of transcriptional regulation that have been understudied in the context of the UPR, such as transcription termination (51), alternative splicing (52), and mRNA stability (53). To investigate these questions and generate a biologically relevant model for probing strictly IRE1-dependent effects, we sought a way to selectively trigger IRE1 in living cells while mimicking its native mechanism of activation. To this end, we developed a method for controlling the oligomeric transition of IRE1 that enables direct, rapid control over IRE1 activity with limited perturbations to the cellular environment. We then used this approach to reveal previously unappreciated aspects of IRE1’s transcriptional program.

## Results

### Optogenetic clustering of IRE1 causes robust IRE1 clustering and activation

**Figure 1:**
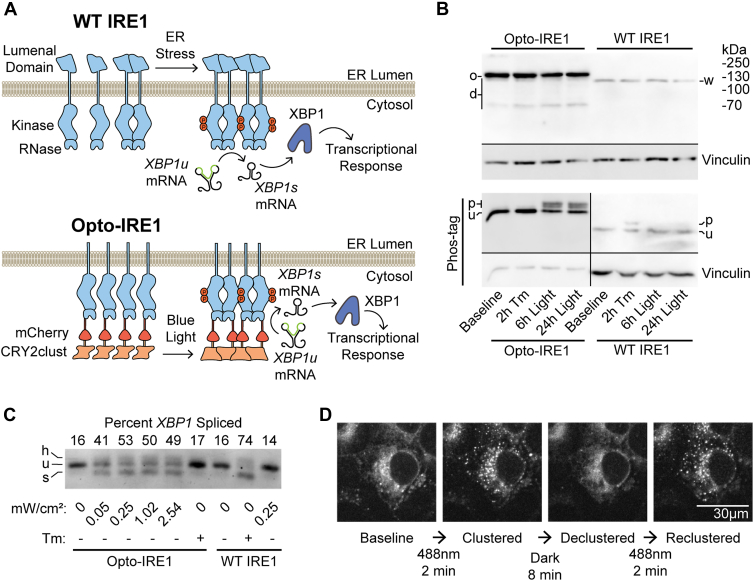
**Development and validation of Opto-IRE1.***A*, diagram of the currently understood mechanism of the activation for WT IRE1 (*top*) and design of our Opto-IRE1 construct (*bottom*). WT IRE1 and Opto-IRE1 form oligomers in response to ER stress or blue light, respectively, and undergo trans-autophosphorylation and activation to splice *XBP1u* mRNA. *B*, anti-IRE1 immunoblots of WT IRE1 and Opto-IRE1 cells treated with tunicamycin (Tm) or light and resolved by SDS-PAGE (*top*) and Phos-tag SDS-PAGE (*bottom*). The WT IRE1 bands are denoted w, the Opto-IRE1 bands are denoted o, and several lower bands are denoted d that likely representing truncations of the 58 kDa CRY2-clust domain. For the Phos-tag gels, the phosphorylated bands are denoted as p, the likely unphosphorylated bands are denoted as u. The contrast and brightness of the Opto-IRE1 and WT sides of the phos-tag blot are separately adjusted to best show detail. Vinculin blots are shown as loading controls. Note that more lysate was intentionally loaded for the WT IRE1 lanes in the Phos-tag blot to compensate for the low signal. See Figure S6 for uncropped blots. *C*, agarose gel depicting an RT-PCR *XBP1* mRNA-splicing assay of Opto-IRE1 and WT IRE1. Labels below the gel indicate treatments of Tm for 2 h or varying irradiances of light for 6 h. Bands corresponding to unspliced *XBP1*, spliced *XBP1*, and their hybrid are denoted with u, s, and h, respectively. The hybrid is believed to be a dimer of the spliced and unspliced PCR products, as it appears only in the presence of both spliced and unspliced XBP1. *D*, timelapse confocal fluorescence microscopy images of Opto-IRE1 cells showing the formation and dissolution of Opto-IRE1 clusters in response to 488 nm blue laser light. IRE1, inositol-requiring enzyme 1.

To enable specific and minimally invasive activation of IRE1 in live cells, we fused the light-inducible clustering domain CRY2clust (54) along with the red fluorescent protein mCherry (55) to the cytosolic C terminus of the human IRE1 protein lacking the lumenal domain (to prevent activation by ER stress). CRY2clust undergoes rapid, reversible homo-oligomerization in response to illumination with blue light, and we reasoned that CRY2clust-mediated control of IRE1 oligomerization may serve as an effective “switch” for IRE1’s enzymatic activities (Fig. 1*A*). Coincidentally, a similar optogenetic IRE1 construct was recently used by Liu *et al.* to study the coordination of IRE1 to stress granules, though its enzymatic functionality and transcriptional effects were not characterized (56). We stably integrated our construct, which we call Opto-IRE1, into the tetracycline-inducible “safe-harbor” locus of U-2 OS Flp-In T-REx cells in which expression of endogenous IRE1 had previously been knocked out (57). Successfully integrated clonal populations were isolated using antibiotic selection followed by fluorescence-activated flow cytometry (FACS) to improve expression homogeneity. This cell line is hereafter referred to as “Opto-IRE1 cells,” while the parental cell line prior to knockout of IRE1 is referred to as “WT cells.”

Immunoblotting for IRE1 showed that expression levels were unchanged by treatment of cells with light or the ER stress–inducing glycosylation inhibitor tunicamycin (Tm) and that Opto-IRE1 expression was one to two orders of magnitude higher than IRE1 levels. Separation of phosphorylation states by Phos-tag PAGE prior to immunoblotting indicated that WT IRE1 becomes phosphorylated in response to tunicamycin and not light (Fig. 1*B*). Inversely, Opto-IRE1 only showed a change in phosphorylation upon light treatment, not tunicamycin, which was expected given that Opto-IRE1 lacks the lumenal “sensor” domain. Next, we tested the *XBP1* splicing activity of Opto-IRE1 cells in response to blue LED light and to ER stress induced by treatment with tunicamycin. In sharp contrast to WT cells, Opto-IRE1 cells exhibited a marked increase in *XBP1* mRNA splicing in response to blue light but not tunicamycin. The lowest light irradiance to induce maximal splicing was ∼250 μW/cm^2^ (Fig. 1*C*), and all further experiments were performed at this irradiance to minimize potential off-target effects of illumination. Opto-IRE1 yielded somewhat lower maximal splicing levels than WT IRE1, which we attribute at least partially to the heterogeneous expression levels of Opto-IRE1 that we observed even after clonal selection and FACS: approximately 28% of cells did not express enough Opto-IRE1 to be visible under confocal fluorescence microscopy (Fig. S1*A*) and thus are likely not exhibiting any IRE1 activity. Despite the very elevated expression level, baseline *XBP1* splicing by Opto-IRE1 was not above that of WT cells, presumably because the absence of the lumenal domain and its binding interfaces greatly decreases the protein’s constitutive propensity for oligomerization and activation. Live-cell spinning-disk confocal microscopy imaging of these cells revealed that Opto-IRE1 could be clustered by illumination with blue light within minutes to form visibly distinct puncta. These puncta fully disappeared several minutes after blue light was turned off, readily reformed in response to subsequent rounds of illumination (Fig. 1*D*), and remained consistently clustered for 24 h under prolonged illumination (Fig. S1*A*), demonstrating the speed, reversibility, and robustness of the process. To validate that the functionality of Opto-IRE1 is not specific to this cell line, we transiently expressed human Opto-IRE1 in previously characterized IRE1α^−/−^/IRE1β^−/−^ double KO mouse embryonic fibroblasts (MEFs) (58) and found that it was able to induce splicing of *XBP1* under light and not tunicamycin (Fig. S1*B*). We also noted that the Opto-IRE1 cell line grew somewhat slower than its WT parental line, and we sought to determine whether Opto-IRE1 activity would change this growth phenotype. We found that sustained blue light treatment of Opto-IRE1 or WT cells for 24 or 72 h did not show a significant change in cell proliferation (Fig. S1*C*), suggesting that IRE1 activation alone is not associated with a dramatic growth phenotype in our model system. Taken together, our results strongly suggest that oligomerization alone is sufficient in mammalian cells to induce both kinase and RNase activation of IRE1.

### Light-induced clustering of Opto-IRE1 activates known IRE1 target genes without triggering general ER stress

**Figure 2:**
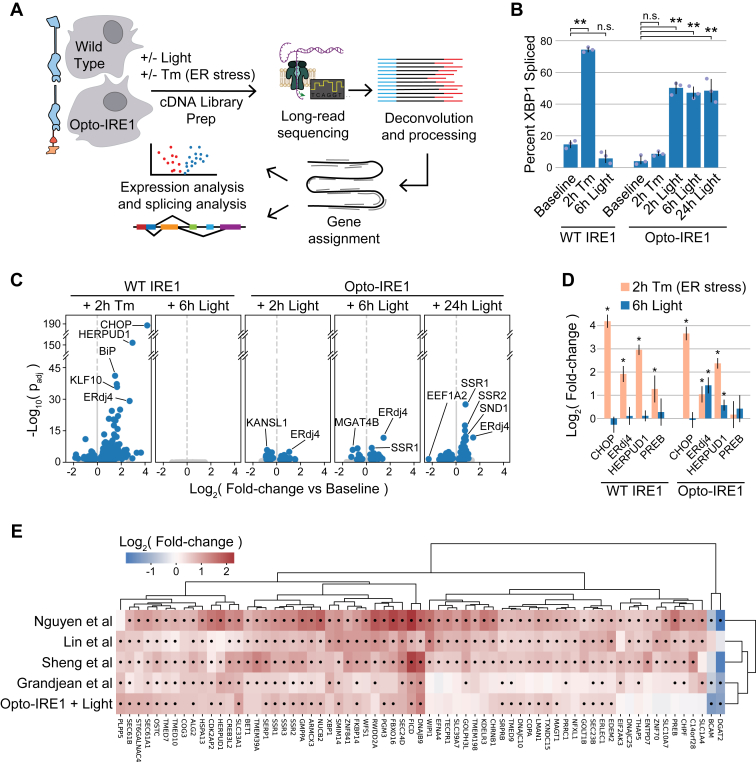
**Opto-IRE1 captures key features of the known IRE1 transcriptional program.***A*, schematic depiction of the transcriptomic experiment and analysis. Cells expressing WT IRE1 or Opto-IRE1 are treated with tunicamycin (Tm) or blue light, and their transcriptomes are sampled with long-read nanopore sequencing. These data are then deconvolved, processed, aligned to the GChr38 reference genome, and analyzed to estimate mRNA expression and splicing. *B*, bar plot showing the *XBP1* splicing percentage within the transcriptomic data for WT and Opto-IRE1 cells at baseline and treated with Tm or light. Error bars correspond to 1 SD (in logit-space) from the mean of three biological replicates. Welch’s *t* test used to find *p*-value, and *p*-values ≤ 0.005 are denoted with ∗∗. *C*, volcano plots showing differential gene expression analysis of WT or Opto-IRE1 cells treated with Tm or light, relative to the untreated samples of the respective cell type. Plots show -log_10_(*p*-value) (y axis) estimated from Wald tests *versus* mean log_2_(fold change) of gene expression relative to baseline expression (x axis) of three biological replicates, and genes with Benjamini–Hochberg FDR-corrected *p*-value ≤ 0.05 are shown in *blue* while others are in *gray*. *D*, bar plot showing log_2_(fold change of gene expression) from the transcriptomic data of four known UPR targets for WT and Opto-IRE1 cells treated with 2 h Tm or 6 h of light. Error bars are shown corresponding to the mean ± 2 standard errors from three biological replicates. *p*-values ≤ 0.05 are denoted with ∗. *E*, heatmap showing the hierarchically clustered log_2_(fold change) of genes across four published IRE1 transcriptomic datasets and our dataset for Opto-IRE1 cells with 24 h light. *Black* dots indicate Benjamini–Hochberg FDR-corrected *p*-value ≤ 0.05, estimated from Wald tests. All datasets contained three replicates. IRE1, inositol-requiring enzyme 1; UPR, unfolded protein response.

**Figure 3:**
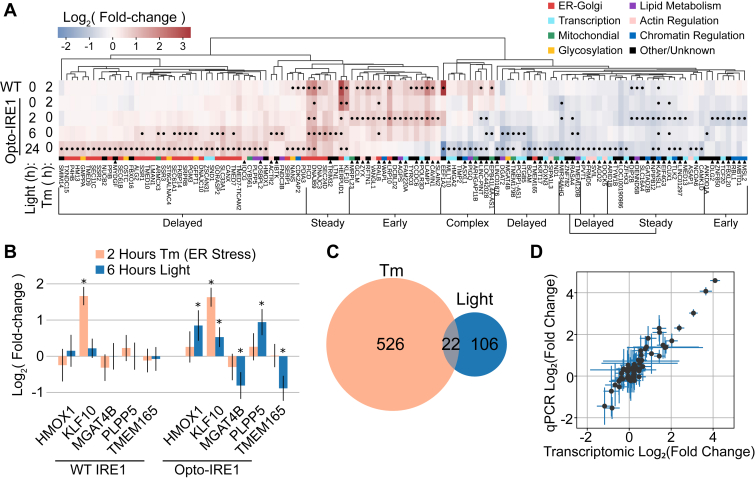
**Opto-IRE1 enables a nuanced and temporally resolved analysis of IRE1 signaling.***A*, heatmap showing hierarchically clustered log_2_(fold change) of genes under treatment of Opto-IRE1 cells with light or tunicamycin (Tm) and of WT cells with Tm, relative to untreated cells of the respective cell type. *Black dots* in the heatmap indicate FDR-corrected *p*-value ≤ 0.05 (estimated from Wald tests), and the genes are clustered on the mean log_2_(fold change) values across three biological replicates. Clusters of genes are qualitatively annotated based on their temporal pattern, and the genes are color-coded based on currently known cellular function. *Black triangles* below the function annotations indicate genes that were not found significant in the datasets we compared against. *B*, bar plot showing log_2_(fold change) of five IRE1 targets for WT cells treated with Tm or Opto-IRE1 cells treated with Tm or light. Error bars are shown corresponding to two standard errors across three biological replicates. Wald-test *p*-values ≤ 0.05 are denoted with ∗. *C*, Venn diagram of the significantly regulated genes shared between Tm (both cell types) and light treatment (only Opto-IRE1). Genes are labeled significant if FDR-corrected *p*-value ≤ 0.05 and log_2_(fold change) ≥ 0.5. *D*, scatter plot showing the correlation between log_2_(fold change) values derived from the transcriptomic data or qPCR assays for 12 genes across seven experimental conditions. Error bars are given as two standard errors across three biological replicates (separate biological replicates for qPCRs and transcriptomics). IRE1, inositol-requiring enzyme 1.

Having validated our ability to selectively stimulate Opto-IRE1’s kinase and RNase activity with light, we set out to map the specific transcriptional effects of isolated IRE1 activation. To this end, we used long-read Oxford Nanopore Technologies sequencing to analyze the transcriptomes of Opto-IRE1 or WT IRE1 cells exposed to light or tunicamycin (Fig. 2*A*). Briefly, RNA was purified from WT or Opto-IRE1 cells, converted to complementary DNA (cDNA) by SMARTer-based reverse transcription with barcoded oligo-dT primers for multiplexing, sequencing adapters ligated, and analyzed using an Oxford Nanopore Technologies PromethION P2 Solo instrument. The resulting reads were basecalled, demultiplexed, aligned to the human reference genome GRCh38.p14, and analyzed for changes in gene expression and splicing using pyDESeq2 (59, 60) (see Experimental procedures for details). To verify that these data capture IRE1 activity, we quantified the splicing of *XBP1* in these data by dividing the number of reads for *XBP1* that lacked the intron region by the total reads that spanned the *XBP1* intron (Fig. 2*B*). This closely matched the PCR analysis in Figure 1*C*: WT IRE1 only responded to tunicamycin, and Opto-IRE1 only responded to light. Differential gene expression analyses were all performed relative only to the baseline (untreated) sample of the same cell type. No cross-analyses were performed between cell types, as the transcriptomic differences between them were found to be much stronger than the changes induced by any treatment (Fig. S2). This may be a result of epigenetic adaptations caused by the long-term lack of IRE1 signaling during the two rounds of clonal selection that separate the Opto-IRE1 and WT cell lines. Analysis of WT cells exposed to light also showed no appreciable changes (Fig. 2*C*), suggesting that 250 μW/cm^2^ blue illumination is a gentle, noninvasive treatment under our cell culture conditions. We primarily focused on the 6- and 24-h light conditions in further experiments due to the stronger transcriptional signatures observed at these times (see Fig. 3 for further elaboration).

Our dataset captured the general signature of the UPR and allowed us to validate transcriptional changes that were previously found to be driven predominantly or partially by IRE1 (Fig. 2*D*): C/EBP homologous protein (CHOP, gene name *DDIT3*), which is known to be affected only by PERK or ATF6 activity (61), was upregulated in both WT and Opto-IRE1 cell lines in response to tunicamycin and not light; ER DNA J domain–containing protein 4 (ERdj4, gene name *DNAJB9*) and homocysteine-inducible ER protein with ubiquitin-like domain 1 (*HERPUD1*), which are known to be upregulated by both IRE1 and ATF6 (30, 62, 63), showed upregulation in response to both light and tunicamycin in both cell lines; and prolactin regulatory element binding (*PREB*), an ER membrane GTP exchange factor found to be upregulated by IRE1 (36, 38, 41, 43, 64), was upregulated only under tunicamycin treatment of WT cells and not any treatment of Opto-IRE1 cells. If we only consider the change in the regulation of *PREB* to tunicamycin between the WT and Opto-IRE1 cells, the data would suggest that it is solely responding to IRE1 activity, which is how several previous studies have connected *PREB* to IRE1. However, the lack of regulation in response to Opto-IRE1 activity instead suggests that *PREB* responds to the combined activity of multiple axes of the UPR, not just IRE1. Understanding this type of coregulation may become possible with precise and specific activation of IRE1, PERK, and ATF6.

### Opto-IRE1 elicits a more focused transcriptional program than other methods

To compare our results to other analyses of the transcriptional effects of IRE1 activity, we examined studies with published transcriptomic datasets in human cells that were experimentally comparable with ours. We focused on four studies that examined IRE1’s transcriptional effects by various means: Nguyen *et al.* infected WT and IRE1-KO cells with OC43 virus (38); Sheng *et al.* (36) and Lin *et al.* (37) used thapsigargin and tunicamycin, respectively, to induce ER stress in WT and *XBP1*-knockdown cells; and Grandjean *et al.* (41) treated cells treated with IXA4, a small molecule found to activate the IRE1–XBP1 signaling axis. We reanalyzed the datasets of these studies, selected substantially upregulated or downregulated genes, and hierarchically clustered them with our dataset for Opto-IRE1 cells with 24 h light (see Experimental procedures for details). Although the IRE1-specific datasets were generated through various methods and in different cell lines, we observed many consistencies between them (Fig. 2*E*). Several well-established targets of IRE1 (such as ERdj4/*DNAJB9*, *FICD*, and signal sequence receptor subunit 1 (*SSR1*) (65, 66)) were significantly upregulated across all IRE1-specific datasets, demonstrating that IRE1 reliably regulates a core transcriptional program across many biological contexts. Many IRE1-sensitive genes, such as *SSR1*, were not upregulated in our shorter 2-h treatments and only became regulated at the 6- and 24-h light timepoints, though they were upregulated in the similarly short treatments of Sheng *et al.* and Grandjean *et al.* This may indicate variation in the speed of IRE1 transcriptional response depending on cell type and method of activation.

Reflecting the activating nature of the XBP1s transcription factor, most genes that showed substantial changes were upregulated. Intriguingly, the only two significantly downregulated genes, diacylglycerol O-acyltransferase 2 (*DGAT2*) and basal cell adhesion molecule (*BCAM*), are two of the most well-reported RIDD target genes (34), and they were unregulated or insignificant in the two studies that are XBP1-specific (Sheng *et al.* and Lin *et al.*). This finding agrees with XBP1-independent regulation of these genes. The dataset clustered closest to ours is that of the Grandjean *et al.* study, which used the small molecule IXA4 to more specifically activate IRE1 activity (41, 42). Many genes, such as *EFNA4* and *NFXL1*, showed little to no expression change in our data and the Grandjean *et al.* data yet showed consistent upregulation in the other three datasets. This may be an indication that some parts of the IRE1 transcriptional program require cooperation of other components of the UPR, since they were only regulated in the datasets that induced the whole UPR.

### Opto-IRE1 reveals temporal regulation and unique transcriptional targets

Since ER stress *in vivo* varies in duration from transient to chronic (67, 68, 69, 70), we sought to investigate how IRE1’s transcriptional effects evolve over our three timepoints. We found 128 genes that appeared to be regulated (false discovery rate-corrected *p*-value ≤ 0.05 and log_2_(fold change) ≥ 0.5) by Opto-IRE1 activation under at least one of our timepoints, and we clustered these genes by their log fold changes in all tunicamycin and light treatments (Fig. 3*A* and Table S1). Nearly all genes were regulated in a consistent manner, with only *EEF1A2* showing major upregulation and downregulation in response to Opto-IRE1 activity. This seems to be an extreme case of divergent crosstalk between the UPR branches, with the IRE1-dependent effect on *EEF1A2* being very delayed. Many genes appeared to be delayed until the 6- or 24-h timepoints, which is potentially a result of chromatin accessibility or regulation by transcription factors downstream from XBP1. Others, such as the GTPase *RALB*, which is thought to mediate ER and Golgi autophagy in connection with cell motility (71, 72), appeared to diminish by 24 h, potentially representing an early, transient signaling pattern. As expected, the upregulated genes were predominantly involved with various ER functions such as protein folding, modification, glycosylation, and translocation, though the downregulated genes were disproportionately related to transcriptional regulation. Some of the earliest downregulated genes were related to epigenetic identity, such as KAT8 regulatory NSL complex subunit 1 (*KANSL1*) (73) and Mbt domain containing 1 (*MBTD1*) (74). This leads us to suspect that IRE1 activity may dysregulate or alter epigenetic labeling, which could help explain observations that IRE1 activity is necessary for cell differentiation of several cell types (46, 47, 48, 49, 75). Broadly, the bias of IRE1 signaling towards upregulation still held true in the temporal analysis, though downregulation became more prominent at the 24-h timepoint. Consistent with the interstudy comparison, these downregulated genes showed more variability across time than the upregulated genes. This could be another indication that IRE1-dependent repression is more variable or sensitive to external factors than upregulation.

Of the 128 genes in Figure 3*A*, 68 did not show changes with false discovery rate-corrected *p*-value ≤ 0.05 in any of the four transcriptomic datasets discussed above (Table S1). These “unique” IRE1 target genes were connected with a variety of cellular functions, including glycosylation, mitochondrial function, and transcriptional regulation. The unique downregulated genes disproportionately featured transcription factors, histone modification proteins, and regulators of epigenetic markers, which represents possible mechanisms for how ER stress has been shown to regulate cell identity (69). Several unique genes were directly connected with ER and Golgi functions: *MGAT4B*, encoding a glycosyltransferase involved in generating branching glycans in the Golgi apparatus (76, 77), and *TMEM165*, encoding a Golgi-resident transmembrane protein thought to regulate calcium and manganese levels in the ER (78, 79), were both downregulated, representing new ways that IRE1 may be regulating glycosylation and ER cation levels, respectively. While it is possible that these genes are artifacts of our specific experimental conditions and cell line, they may have also been identified because inducible oligomerization of IRE1 mimics its endogenous activation in a way that was not fully captured by previous approaches.

### The Opto-IRE1 transcriptional program is reproducible and differs from activation by tunicamycin

Several genes appeared to exhibit a UPR-repressed pattern, wherein the regulation induced by Opto-IRE1 activation at 2 h was not observed under activation of the whole UPR with tunicamycin for 2 h in WT cells: *MRPL23*, *SLAIN2*, *MSL2*, and *MBTD1*. These genes were all regulated almost exclusively by Opto-IRE1, with no significant change in expression during tunicamycin treatment of WT cells (Fig. 3*B*). Several genes (labeled “Complex”) also showed significant upregulation under tunicamycin and significant downregulation under Opto-IRE1, such as *EEF1A2*, an isoform of the alpha subunit of the ribosomal elongation factor 1 complex which facilitates delivery of aminoacyl tRNA to translating ribosomes (80). We suspect that this counter-regulation is a result of delayed repressive effects of a downstream IRE1 target overriding activating effects of an early UPR component. While the 2-h tunicamycin treatment in WT cells was satisfyingly similar to the 2-h light stimulation of Opto-IRE1 cells, it was also strikingly similar to tunicamycin treatment of Opto-IRE1 cells, despite the apparent lack of IRE1 activation. In fact, several genes showed nearly identical regulation at 2 h between isolated IRE1 activity (Opto-IRE1), full UPR activity (WT + tunicamycin), or UPR activity without IRE1 (Opto-IRE1 + tunicamycin). For example, *RALB* appeared to exhibit a nonadditive response to the different axes of the UPR, which is a potential cause for why it was not identified in studies comparing ER stress with and without the IRE1–XBP1 axis. All three timepoints of light treatment only share 22 significant genes with tunicamycin treatment of both cell types (Fig. 3*C*), which is almost certainly due to the fact that tunicamycin induces a much more severe, cell-wide response than isolated IRE1 activity. Overall, these results further illustrate the complexity of the UPR and the nuanced regulation that interferes with isolating the effects of each sensor protein.

To validate some of our results with an orthogonal approach and verify the robustness of our sequencing data analysis, we performed real-time quantitative polymerase chain reaction (qPCR) assays on biological replicates of the experimental conditions used for transcriptomics. For this, we selected the four known targets of UPR regulation from Figure 2*C*, the four UPR-repressed genes discussed above, *XBP1*, *SEC23A* (SEC23 homolog A, encoding a close paralog of the known IRE1 target *SEC23B*), *TAPBP* (TAP-binding protein, a component of the protein loading complex (81) and a reported RIDD target (82)), and *KLF10* (Kruppel-like factor 10), a repressor of TGF-β signaling and another known target of UPR regulation (83)). Overall, our qPCR results agreed remarkably well with our transcriptomic results (Figs. 3*D* and S3), indicating that Opto-IRE1 induces a reproducible transcriptional program across biological replicates and experimental methods that differs notably from the activation of IRE1 with tunicamycin.

### The XBP1-independent effects of Opto-IRE1 are limited and primarily related to ER function

**Figure 4:**
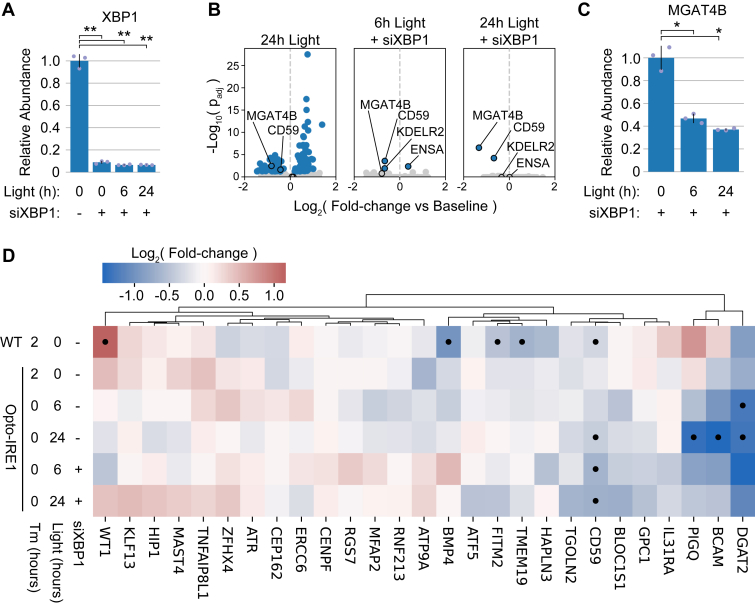
**Analysis of XBP1-independent transcriptional effects of Opto-IRE1.***A*, bar plot showing relative qPCR expression levels of *XBP1* in Opto-IRE1 cells treated with light or anti-*XBP1* siRNA (siXBP1). Shown as mean ± 1 SD across three biological replicates. Welch’s test *p*-values ≤ 0.005 are denoted with ∗∗. *B*, volcano plots showing differential gene expression analysis of Opto-IRE1 cells treated with siXBP1 and varying doses of light. The 24 h light treatment is relative to the untreated cells, while the siXBP1 + light treatments are relative to cells treated only with siXBP1. Plots show log_10_(*p*-value) (y axis) estimated from Wald tests *versus* mean log_2_(fold change) of gene expression (x axis) across three biological replicates. Genes with a Benjamini–Hochberg FDR-corrected *p*-value ≤ 0.05 are shown in *blue* while others are in *gray*. Select genes are circled and annotated. *C*, bar plot showing relative qPCR expression levels of *MGAT4B* in Opto-IRE1 cells treated with light and siXBP1. Shown as mean ± 1 SD across three biological replicates. Welch’s test *p*-values ≤ 0.05 are denoted with ∗. *D*, heatmap showing the hierarchically clustered log_2_(fold change) of previously reported RIDD target genes in WT or Opto-IRE1 cells treated with Tm, light, or siXBP1. All samples are relative to their respective untreated cells, though siXBP1 conditions are relative to Opto-IRE1 cells treated only with siXBP1. *Black* dots in the heatmap indicate Benjamini–Hochberg FDR-corrected Wald-test *p*-value ≤ 0.05. IRE1, inositol-requiring enzyme 1; RIDD, regulated IRE1-dependent decay.

In parallel with the *XBP1* signaling axis, IRE1 is capable of cleaving a range of other RNA targets, leading to their eventual degradation, in a process termed RIDD (32, 33, 34, 84, 85). RIDD appears to serve distinct functions in a cell type– and context-dependent manner, including lysosome repositioning (86) and maternal mRNA decay during embryogenesis (87). To isolate the transcriptomic effects of Opto-IRE1 from those of XBP1, we utilized siRNA to knock down *XBP1* mRNA by approximately 14-fold in Opto-IRE1 cells while activating these cells with light (Fig. 4*A*). A control siRNA treatment also showed no effect on *XBP1* splicing or expression, indicating that the *XBP1* siRNA treatment was effective and specific (Fig. S1, *D* and *E*).

We found that the direct effects of Opto-IRE1 activity appeared to be extremely limited relative to the effects of Opto-IRE1 with normal XBP1 expression: only *CD59*, KDEL receptor 2 (*KDELR2*), endosulfine Alpha (*ENSA*), and *MGAT4B* appeared to be substantially affected by the activation of Opto-IRE1 with an FDR-corrected *p*-value ≤ 0.05 (Fig. 4*B*). Given that *ENSA* is upregulated, it would necessarily be a downstream effect rather than a direct target of IRE1’s RNase, which leaves only the three downregulated genes as potential IRE1 substrates. While *CD59* is a commonly reported RIDD target, *MGAT4B* and *KDELR2* have not previously been associated with RIDD and represent novel potential IRE1 RNase substrates. Intriguingly, both genes encode ER-related proteins, suggesting that RIDD in this case may be focused on the ER. As mentioned previously, *MGAT4B* encodes an ER-Golgi glycosyltransferase that is critical for creating branching glycans (76, 77), indicating that RIDD could alter glycosylation patterns by repressing *MGAT4B* (Fig. 4*B*). Although *MGAT4B* fell short of statistical significance at 6 h light in the transcriptomic data, qPCR of *MGAT4B* showed that the repression is also present at that timepoint (Fig. S3). Furthermore, *MGAT4B* is also significantly downregulated in the light-treated Opto-IRE1 conditions without siRNA and contains two predicted recognition sites for the IRE1 RNase (Fig. S4). *KDELR2* is one of the several genes that recognize the KDEL sequence of ER-resident proteins in order to retain them in the ER (88, 89), potentially indicating that RIDD could alter the balance between KDEL receptors and thus the preference to retain certain KDEL sequences (90). *KDELR2* fell below the significance threshold at 24 h of light and had a more limited fold change, which may indicate that it is only targeted by RIDD for a short time. However, the fact that *KDELR2* is not regulated under the original 24-h condition also suggests that it may be an artifact of the analysis, and it also only contains a weak hairpin site for the IRE1 RNase (Fig. S4). Since the *XBP1* knockdown experiment would also capture any transcriptional effects of the IRE1 kinase domain outside of trans-autophosphorylation (91), these limited results also suggests that the kinase domain did not induce strong transcriptional changes.

We then sought to check whether previously reported RIDD targets were downregulated in any of our data, so we clustered the log_2_(fold changes) of genes reported to be XBP1-independent in the Le Thomas *et al.* and Quwaider *et al.* studies (Fig. 4*D*) (34, 85). Few of these genes were notably downregulated under any condition, and only three genes were downregulated by 2-fold or more in either of the siXBP1 conditions: *DGAT2, BCAM*, and phosphatidylinositol glycan anchor biosynthesis class Q (*PIGQ)*. Two of these genes, *DGAT2* (92, 93) and *PIGQ* (94), are related to lipid metabolism in the ER, while BCAM (95) is not strongly associated with ER function. While several other genes (such as *BMP4*, *HAPLN3*, and *TMEM19*) appeared to be downregulated under tunicamycin treatment of WT cells, they did not appear to be affected by light under siXBP1 treatment, indicating that these may be regulated by XBP1 rather than directly by RIDD. Although the partial dependence of RIDD on translational inhibition by PERK could explain the lack of downregulation under Opto-IRE1 (33), the overall lack of downregulation of these commonly reported RIDD targets under tunicamycin treatment of WT cells is puzzling and suggests a generally low level of RIDD activity in our U-2 OS cell line. Combined with reports that the substrate preference of RIDD varies depending on many cellular contexts, including cell type, oligomeric state of IRE1, and PERK interaction (32, 33, 34, 87), these data suggest that RIDD overall in this cell line is a limited phenomenon that targets a select set of primarily ER-related gene transcripts for degradation.

### ER stress regulates levels of alternatively spliced mRNA isoforms

**Figure 5:**
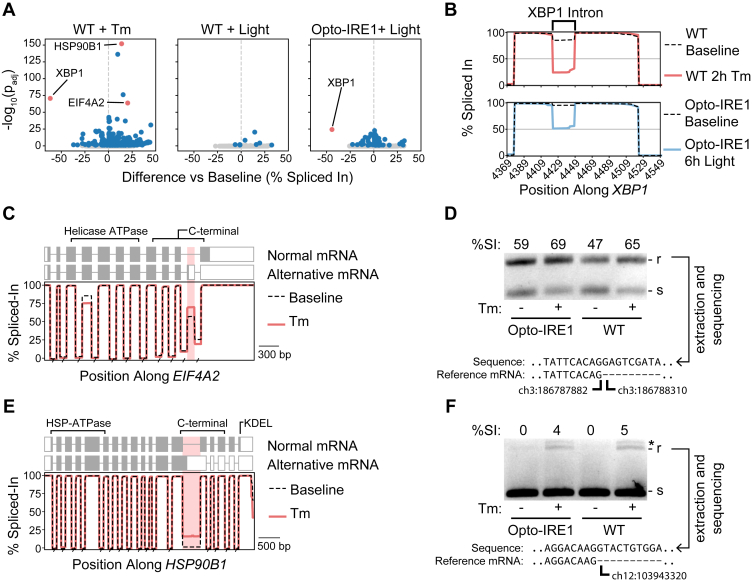
**Detection of UPR-dependent alternative splicing.***A*, volcano plots showing the mean difference in percent-spliced-in of exons across three biological replicates (x axis) *versus* the -log_10_(Benjamini–Hochberg FDR-corrected *p*-value, Welch’s *t* test) (y axis) for WT cells treated with 2 h of tunicamycin (Tm) or 6 h of light and Opto-IRE1 cells treated with 24 h of light, relative to their respective untreated cells. *B*, line plot showing the mean percent-spliced-in (y axis) *versus* the genomic position (x axis) along *XBP1* reads near the unconventional intron for WT cells and Opto-IRE1 cells treated with Tm or light, respectively. *C* and *E*, line plot showing mean percent-spliced-in (y axis) *versus* the genomic position (x axis) along *EIF4A2* reads (*C*) or *HSP90B1* (*E*) for WT IRE1 cells treated with Tm. The normal and alternative mRNA transcripts are shown above the plots with boxes indicating exons and the shaded areas indicating the translated reading frame. The known functional protein domains are annotated above these structures. *D* and *F*, agarose gel depicting the products of a PCR across the alternatively spliced region of *EIF4A2* (*D*) or *HSP90B1* (*F*) for WT and Opto-IRE1 cells treated with and without Tm (separate biological replicates from those used for panels *A*–*C*, and *E*). The larger, intron-retaining product is denoted with “r,” the smaller spliced with “s,” and off-target products with an asterisk. The sequence of the larger product is shown below, aligned to the 5′ splice junction. IRE1, inositol-requiring enzyme 1; UPR, unfolded protein response; EIF4A2, eukaryotic initiation factor A2.

Since splicing is a critical component of IRE1’s signaling pathway, we wondered whether any other unexpected splicing changes occur under ER stress. After aligning transcripts against the reference genome, we calculated the rate at which each base was retained in the transcriptome as a percent-spliced-in value (see Experimental procedures for details) and then compared this value between samples to identify differentially spliced regions. We excluded the highly variable aspects of alternative transcription initiation and termination from this analysis, since this method is best suited to investigating binary inclusion/exclusion of transcript regions. In Opto-IRE1 cells, we did not observe any light-induced splicing changes to nearly the same extent as the *XBP1* intron, supporting the common conclusion that *XBP1* is the only transcript that is robustly spliced by IRE1 (Fig. 5*A*). In contrast, tunicamycin-mediated induction of ER stress strongly altered splicing of many regions, including (as a satisfying internal control) the nonconventional intron of *XBP1* (Fig. 5*B*). We selected two promising alternatively spliced genes to investigate further: eukaryotic initiation factor A2 (*EIF4A2*) encodes one of two isoforms of a key subunit of the eukaryotic initiation factor 4F complex (96, 97, 98), and heat shock protein 90 B1 (*HSP90B1*) is a highly-abundant ER-resident chaperone that plays key roles in shuttling of certain proteins through the ER, notably including many immune response membrane proteins (99, 100). For *EIF4A2*, we found that an optional exon was being retained from intron 10 under tunicamycin-induced stress (Fig. 5*C*), which we verified by PCR and Sanger sequencing across the splice boundary (Figs. 5*D*, S5). This exon introduces a premature stop codon that interrupts the C-terminal domain and prevents proper helicase activity, and studies report that the transcript containing this exon is recognized and degraded by the nonsense-mediated decay (NMD) pathway (101, 102). However, the truncated protein appears to be expressed in some cell types (103, 104), albeit short-lived due to ubiquitylation. While the truncated protein does not appear to be incorporated into the translation initiation complex, the alternative transcript may play a biologically relevant role in the regulation of EIF4A2 protein expression levels by NMD (102). For *HSP90B1*, we found that tunicamycin treatment increased retention of intron 13, which also results in the introduction of a premature stop codon. The resulting 661aa-long truncated protein, if expressed, would retain the most critical domains for HSP90 chaperone function (105, 106), though it would lack the KDEL ER-retention signal and the C-terminal domain that is thought to regulate dimerization and cochaperone interactions (107, 108). While this could result in a monomeric form that is exported to the cytosol, its functionality outside of its normal dimeric form is unknown. This alternative splicing event may also be a cotranscriptional mechanism for control over *HSP90B1* expression. The fact that these changes are not present under Opto-IRE1 activation but are present under tunicamycin treatment regardless of cell type suggests that they are not dependent on IRE1 activity, instead likely a result of ATF6 or PERK signaling.

### The UPR predominantly alters RNA isoform abundances independently of NMD inhibition

**Figure 6:**
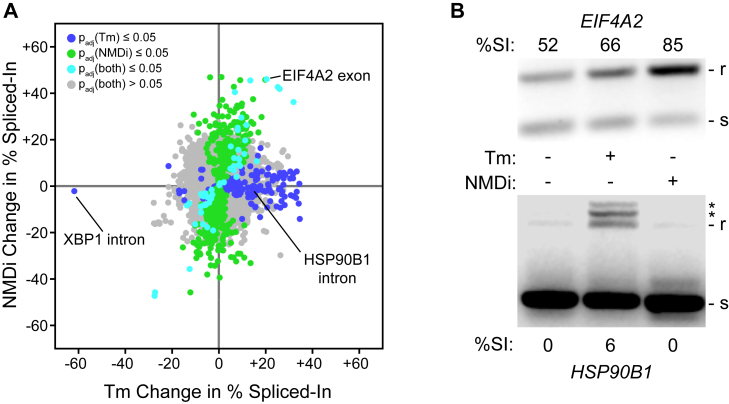
**Splice isoform changes induced by the UPR are largely distinct from those seen under NMD inhibition.***A*, a plot of mean changes in percent spliced in across three biological replicates paired between treatment with tunicamycin (Tm) and an inhibitor of NMD (NMDi), relative to untreated cells. Each point is an intron/exon region, and its color corresponds to the Benjamini–Hochberg FDR-corrected *p*-value for both treatments, calculated with Welch’s *t* test. *B*, agarose gel depicting the products of a PCR across the alternatively spliced region of *EIF4A2* (*top*) or *HSP90B1* (*bottom*) for WT cells treated with Tm or NMDi (separate biological replicates from those used for panel *A*). The larger, intron-retaining product is denoted with “r,” the smaller spliced with “s,” and off-target products with an asterisk. UPR, unfolded protein response; EIF4A2, eukaryotic initiation factor A2.

The possibility of the *EIF4A2* and *HSP90B1* transcripts being NMD targets is supported by evidence that PERK activity suppresses the NMD pathway and creates higher levels of NMD-targeted transcripts (109), which would also result in the accumulation of the alternate transcripts. To gain more insight into the ability of the UPR to alter RNA isoform abundance, we wanted to determine what fraction of our observations in Figure 5 were due to the previously documented inhibition of NMD by PERK (109). To do so, we treated WT cells with the SMG1 inhibitor 11j (hereafter NMDi) (110) and analyzed the resulting transcriptome. This analysis revealed that although some of the changes in RNA isoform abundance seen under tunicamycin treatment can be replicated by NMDi, the two treatments are remarkably distinct (Fig. 6*A*). The NMDi treatment appears to generate more changes in isoform abundance than tunicamycin does. Intriguingly, the effects of UPR induction appear to be strongly biased towards retaining RNA regions, whereas the NMDi treatment is relatively unbiased. This may indicate that the UPR selectively alters splicing in a manner that encourages sequence retention. The change in isoform abundance observed for *EIF4A2* appears to be a result of NMD inhibition, as NMDi replicates and exceeds the splicing pattern seen under tunicamycin treatment (Fig. 6*B*). However, the change observed for *HSP90B1* does not seem to be NMD-dependent, as NMDi did not replicate the increase in retention seen under tunicamycin treatment. This observation suggests either a true change in alternative splicing or an altogether different mechanism of RNA regulation by the UPR.

## Discussion

As a central component of a critical stress response pathway, mammalian IRE1 signaling has been the subject of numerous investigations. While many core components of the IRE1 transcriptional program are generally reproducible and agreed upon, the full extent and temporal progression of IRE1 signaling remain unclear. Major open questions that still evade consensus include the extent of RIDD and the potential interplay between the UPR and mRNA quality control pathways, such as NMD. Moreover, the variation in downstream effects of IRE1 signaling across cell types, tissues, and developmental stages remains poorly understood. Thus, the development of a minimally invasive, easily portable, and highly specific way to induce IRE1 signaling with precise spatiotemporal control promises to greatly enhance our understanding of the UPR’s molecular wiring. The landmark works by Grandjean *et al.* and Madhavan *et al.* to develop and characterize the small molecule activator of IRE1, IXA4, made important strides towards filling this gap (41, 42). As demonstrated in the present study, Opto-IRE1 exhibits minimal basal activity and does not respond to ER stress due to the absence of its lumenal domain, making it a perfectly orthogonal “switch” that can be integrated into a living system and activated on demand. While small-molecule activation carries obvious pharmacological advantages, our complementary approach in Opto-IRE1 has little to no detectable off-target activity, can be applied with subcellular spatial precision, and is rapidly reversible on the timescale of a few minutes. Opto-IRE1 also robustly induces the protein’s *trans-*autophosphorylation, suggesting that this system can be used to separately investigate the downstream cellular functions of IRE1’s kinase (*e.g.* by expressing a catalytically dead RNase mutant of Opto-IRE1) (91). Since optogenetic control is compatible with model systems from *Caenorhabditis elegans* to zebrafish and mice (111, 112) and we demonstrated Opto-IRE1’s cross-species functionality in MEFs, Opto-IRE1 thus offers a powerful way to study the downstream effects of pulses of isolated IRE1 activation at a precise location and time in a developing or adult organism.

With Opto-IRE1, we have demonstrated that IRE1 signaling exhibits a temporally evolving progression with the earliest regulated genes being related to transcription, cell identity, and ER function. This is followed by the regulation of many more genes primarily involved in ER function, as well as diverse cellular processes such as lipid metabolism, mitochondrial function, and mRNA regulation. Many of these delayed proteins are likely downstream results of the early elements in the IRE1 pathway, though they may also be inhibited by chromatin accessibility or competing transcription factors. This dynamic regulation is accompanied by early and steady regulation of the most well-known IRE1 targets, such as ERdj4/*DNAJB9* and *HERPUD1*. We have also identified new targets of IRE1 regulation, expanding the known IRE1 transcriptional program. For example, repression of *TMEM165* indicates a potential mechanism for IRE1-mediated control over Ca^2+^ and Mn^2+^ levels in the ER, and repression of *MGAT4B* indicates a new putative mechanism for UPR-mediated modulation of glycosylation. Nearly all of the genes downregulated by Opto-IRE1 in this analysis were not present in the four datasets we compared ours against, which could be a result of several factors: the various experimental methods used between this study and those we re-analyzed could have preferentially captured different gene targets; alternatively, these genes may not be expressed at measurable levels in the cell lines used by the other studies, thereby preventing observation; finally, direct activation of IRE1 by inducible oligomerization may capture its endogenous targets in a way that was inaccessible in earlier work.

Beyond the regulation of gene expression, we also observed ER stress-dependent changes in the abundance of transcript isoforms of several genes, which may result in altered protein levels or functions. These changes appeared to be entirely independent of IRE1 and only some can be explained as PERK-mediated inhibition of NMD, as they were replicated by treatment with an NMD inhibitor. However, many transcript regions were uniquely regulated by ER stress, potentially representing a new axis by which the UPR regulates protein expression. This could take many forms, such as changes in mRNA quality control in the cytoplasm or altered splicing in the nucleus and deserves further investigation.

While RIDD has been heavily investigated, the affected transcripts vary substantially among studies. Additionally, RIDD has been proposed to be dynamically regulated by many factors, including PERK activity and the oligomeric state of IRE1 (33, 84). Because of this complexity, the selectivity and control offered by Opto-IRE1 is well-suited to exploring RIDD and other potential XBP1-independent effects of IRE1 activity. Isolated from the activities of the UPR and XBP1, we only detected the direct downregulation of four previously identified RIDD targets, *DGAT2*, *BCAM*, *PIGQ*, and *CD59* (34) as well as two novel targets *MGAT4B* and *KDELR2* (Fig. 4). Although the exact oligomeric state of Opto-IRE1 is unknown, we suspect that the rapid dynamics of CRY2clust (54) create a mixed population of states that would be competent for various types of RIDD activity (34). Furthermore, RIDD has been shown to be stronger when IRE1 is overexpressed (33, 113, 114), likely due to the resulting increased stoichiometry between the RNase domain and cytosolic mRNA. While our data demonstrates Opto-IRE1’s ability to engage with RIDD targets, it also suggests that RIDD in the U-2 OS cell line and under our cell culture conditions is limited to a select few genes. Porting Opto-IRE1 to a model system with more prominent RIDD activity may yield important insights about this mode of IRE1 activity in the future.

The new targets of IRE1 regulation that we identified belong to a variety of functional categories, such as transcriptional regulation, lipid metabolism, actin remodeling, and ER homeostasis. Many of the observed downregulated genes, such as *KANSL1* (73) and *MBTD1* (74), were related to epigenetic regulation and chromatin remodeling, indicating that IRE1 may be able to alter a cell’s epigenetic character. This could be a mechanism for cells to adapt to prolonged ER stress and may hint at an explanation for IRE1’s control over certain types of cell differentiation (46, 47, 48, 49), though this possibility demands further investigation. We have also further characterized the temporal evolution of IRE1 signaling and showed that the bulk of the transcriptional program is delayed for several hours after IRE1 is activated. Such a delay may serve to buffer the cell’s response to low-level ER stress that does not warrant the entirety of IRE1’s transcriptional effects. Consistent with other studies, our findings place IRE1 at a complex nexus of intracellular signaling, further illustrating the importance of investigating IRE1 activity with precise next-generation approaches.

## Experimental procedures

### Cell culture and experimental reagents

All U-2 OS and MEF cells were grown at 37 °C with 100% humidity and 5% CO_2_ in either Dulbecco’s modified Eagle Medium (DMEM, Gibco) or Fluorobrite DMEM (Gibco), both supplemented with 10% fetal bovine serum, 2 mM L-glutamine, and 100 units/ml penicillin/streptomycin. Where stated, cells were treated with dimethyl sulfoxide (DMSO)-solubilized tunicamycin (Sigma) to 5 μg/ml, DMSO-solubilized doxycycline (Sigma) to 300 nM, DMSO-solubilized SMG1 inhibitor 11j (Arctom. CAS NO. 1402452-15-6) to 0.5 μM, or blue light treatment with a custom illuminator outputting 250 μW/cm^2^. WT cells that did not receive tunicamycin of 11j instead received an equivalent dosage of DMSO. All cell lines used in the study tested negative for *mycoplasma* contamination when assayed with the Universal *Mycoplasma* Detection Kit (ATCC 301012K) (Fig. S6*B*).

### Generation of the Opto-IRE1 cell line

Opto-IRE1 was expressed in an IRE1-null background using the Flp-In system. The expression plasmid (Addgene plasmid ID 242429) encoding (delta-lumenal domain)IRE1-mCherry-CRY2clust in the pcDNA5/FRT/TO backbone (Thermo Fisher Scientific V652020) was stably integrated into IRE1 knock-out U-2 OS Flp-In T-Rex cells that were generated in an earlier study (PWM254) (57). To achieve stable integration, parental cells were plated into wells of a 6-well plate at a density of 1.7e4 cells/cm^2^. The following day, they were simultaneously transfected with 1.7 μg of the Flp recombinase expression vector, pOG44 (Thermo Fisher Scientific V600520), and 300 ng of the IRE1-mCherry-CRY2clust expression plasmid. Transfections were carried out in antibiotic-free DMEM, using the Fugene HD transfection reagent (Promega E2311) and following the manufacturer’s protocol. Twenty-four hours post transfection, cells were split 1:6 into 10-cm dishes and allowed to adhere and recover for an additional 24 h. The growth medium was then supplemented with 150 μg/ml hygromycin B (Thermo Fisher Scientific 10687010) to initiate selection. Cells were maintained in selection medium until single clones became clearly visible and reached ∼3 mm in diameter. Three clones were picked using sterile cloning cylinders (Corning 3166-10), expanded, and assayed for doxycycline-inducible expression of the recombinant construct. The clone that showed the most robust increase in IRE1-mCherry-CRY2 expression upon addition of doxycycline was expanded further and sorted on a Becton Dickinson FACSAria III sorter, selecting for cells with “low” or “high” levels of mCherry fluorescence relative to the parental PWM254 cell line (Fig. S7). The “low” pool of sorted cell cells was assigned the identifier “cVBL001,” confirmed to be free of *mycoplasma* contamination by PCR (30-1012K, ATCC) and used as the “Opto-IRE1” cells for subsequent experiments in this study.

### Light stimulation of cells

A custom illuminator was created to fit 24-well plates using 3.2 V blue LEDs (peak wavelength of 470 nm, 50% power angle of 30°, and brightness of 4.8 cd) mounted to a printed circuit board (PCB) such that each LED is centered on a well. These LEDs were controlled by a Metro 328 Arduino with PCB-mounted shift registers and run at 5% duty in a 1 s cycle to achieve the 250 μW/cm^2^ irradiance, as measured by a laser power meter. The schematics of the PCBs and the code is available in the Zenodo repository, and all electrical part numbers can be found in the Reagents and Materials file.

### Microscopy of Opto-IRE1

Cells were plated in collagen-coated glass-bottom tissue culture dishes and incubated for 24 h. These were then treated for 24 h with supplemented Fluorobrite with doxycycline. For the live-cell imaging in Figure 1*D*, confocal imaging was carried out on a Nikon Ti-E inverted microscope equipped with a Yokogawa CSU-X high-speed confocal scanner unit and a pair of Andor iXon 512 × 512 EMCCD cameras. Images were obtained using a 100 × 1.49 NA oil immersion objective. The following filters were used in the microscope: Di01-T405/488/568/647 quad-band dichroic (Semrock) and ET607/52m emission filter (Chroma). All components of the microscope were controlled by the μManager open source platform (115). The microscope stage was enclosed in a custom-built incubator that maintained preset temperature and CO2 levels for prolonged live-imaging experiments. For the fixed cell images in Figure S1*A*, cells were fixed with a warm solution of pH 7.5 PBS with 4% paraformaldehyde and 0.2 M sucrose for 10 min, followed by three washes with warm PBS, then covered with PHEM buffer (60 mM Pipes, 25 mM Hepes, 10 mM EGTA, 2 mM MgCl2) and stored at 4 °C. Prior to imaging, the cells were permeabilized with 0.1% Triton X-100 in warm PBS for 5 min, followed by a 3 min incubation with 300 nM DAPI stain in PBS. The cells were then washed three times and covered with PBS. Scanning confocal imaging was carried out on a Nikon Ti A1R inverted microscope using a 60x 1.4 NA oil immersion objective. The following filters were used in the microscope: 405/488/561/640 quad-band dichroic, 450/50 (for the DAPI channel), and 595/50 emission filter (for the OptoIRE1 mCherry channel). Images were acquired with 8.3s exposure.

### RNA purification

For all experiments using RNA, cells were seeded into a black 24-well glass-bottom plate at 20,000 cells per well and incubated for 24 h in supplemented DMEM, then for 24 h in supplemented Fluorobrite medium with doxycycline. Cells were then given treatments of light, tunicamycin, or SMG1 inhibitor 11j for prescribed durations and irradiances before being washed with PBS and lysed with Trizol (Thermo Fisher Scientific). 20% V/V chloroform was added and RNA was then purified from the aqueous layer using a spin-column kit (RNA Clean & Concentrator-5, Zymo Research #R1015) according to manufacturer protocol and quantified using a Nanodrop One C (Thermo Fisher Scientific).

### siRNA treatment

Opto-IRE1 cells were electroporated with 5 μM anti-XBP1 siRNA (Santa Cruz Biotechnology, sc-38627) or 5 μM scrambled control siRNA-A (Santa Cruz Biotechnology, sc-37007) with a Neon N x T electroporator (Thermo Fisher Scientific) set to 1230V, 10 ms, and 4 pulses. These cells were then plated for RNA experiments as described above, but with 40,000 cells per well to account for roughly 50% mortality after electroporation.

### Transcriptomic prep

RNA was collected as described above in biological triplicate and converted to double stranded cDNA using an adaptation of SMARTer-based protocol (116). Two hundred nanograms of total RNA in 11.5 μl water was mixed with 1 μl of 10 μM barcoded poly-dT primer and 1 μl of 10 mM dNTPs before being incubated at 65 °C for 5 min, 4 °C for 5 min, and 42 °C for 2 min. The reaction was then quickly mixed with 0.5 μl RNaseOUT (Thermo Fisher Scientific #10777019), 1 μl of 20 mM strand-switching primer, 1 μl Maxima H Minus Reverse Transcriptase (Thermo Fisher Scientific EP0751), and 4 μl Maxima H Minus buffer. This reaction was incubated at 42 °C for 90 min before being heat killed at 85 °C for 5 min and cooled to 4 °C. This was PCR amplified by combining 2 μl of cDNA, 2 μl Platinum SuperFi II DNA Polymerase (Invitrogen # 12361010), 20 μL Platinum SuperFi II buffer, 2 μl of 12 μM PR2 primer, 2 μl 10 mM dNTPs, and 72 μl of water and then thermally cycled using the following program: 98 °C for 30s, 16×[98 °C for 10s, 60 °C for 10s, 72 °C for 6 min ], and held at 4 °C. An equal volume of sparQ PureMag Beads was then mixed with the PCR reaction containing amplified cDNA, mixed for 5 min, separated from supernatant on a magnet, washed twice with 500 μl of 70% ethanol, lightly dried, resuspended in 21 μl water, mixed for 10 min, and separated with a magnet. The supernatant containing double stranded cDNA was then quantified by fluorescence using the Quant-iT Picogreen reagent (Thermo Fisher Scientific P7589), and equal masses of DNA were pooled from samples with unique molecular barcodes to a total of 150 ng of multiplexed DNA and diluted to 50 μl. Three microliters of enzyme and 7 μl of buffer from the NEBNext Ultra II End Repair/dA-Tailing Module (New England Biolabs E7546S) were added to the DNA pool and incubated at 20 °C for 5 min and 65 °C for 5 min. End-repaired DNA was then mixed with 60 μl sparQ PureMag Beads, mixed for 5 min, separated from supernatant on a magnet, washed twice with 200 μl 70% ethanol, lightly dried, resuspended in 60 μl water, mixed for 10 min, and separated with a magnet. The end-repaired cDNA in the supernatant was then ligated to sequencing adapters by adding 25 μl ligation buffer and 5 μl ligation adapter from Ligation Sequencing Kit V14 (Oxford Nanopore Technologies SQK-LSK114) as well as 10 μl Quick T4 Ligase (New England Biolabs E6056S) before incubation for 10 min at room temperature. Forty microliters of sparQ PureMag Beads was then added to the ligated DNA library, mixed for 5 min, separated from supernatant on a magnet, washed twice with 250 μl of short fragment buffer, lightly dried, resuspended in 32 μl elution buffer (Oxford Nanopore Technologies SQK-LSK114), mixed for 10 min, and separated with a magnet. The resulting supernatant containing the prepared DNA library was then loaded onto PromethION Flow Cell R10.4.1 (Oxford Nanopore Technologies FLO-PRO114M) as per manufacturer protocol and sequenced for 72 h.

### Transcriptomic data analysis

Sequencing traces were basecalled using Dorado v0.9.6 with the dna_r10.4.1_e8.2_400 bps_sup v5.0.0 model. The resulting pooled reads were assigned to samples by detecting the barcode sequences of the VN primers: This was done by first searching for the poly-T region of the VN primer and the common primer sequence, then searching near those features for the unique sequences. Barcodes were confidently assigned when a unique sequence was matched with three or fewer mismatches in the correct direction relative to the poly-T region and/or the common sequence, and reads were excluded from further analysis if multiple or no barcodes were successfully assigned. The edlib package was used for all barcode alignments (117). Any identified barcode, poly-T, and common primer sequences were trimmed, and the remaining internal sequence was aligned to the human reference genome GRCh38.p14 (Genome Research Consortium), excluding revised scaffold contigs, using the minimap2 package set to the “splice” preset (118). The resulting alignments were each assigned to a gene by finding the annotation in the GRCh38 gene file with the greatest ratio of overlapping sequence to the length of the annotation or alignment, whichever is longer. These gene assignments were then used to generate per-sample gene counts, and these counts were analyzed for differential gene expression.

We analyzed the data of our study and those we compared against using the pyDESeq2 implementation of DESeq2 (59, 60). For these analyses, each combination of cell type and treatment was treated as a separate experimental condition, the min_mu parameter was set to 15, and all other parameters were kept as the default values. Genes were selected as “significant” if they had FDR-corrected *p*-values (Benjamini–Hochberg) ≤ 0.05 and |log_2_(fold change)| ≥ 0.5. For hierarchical clustering in heatmaps, we used Ward’s method.

### Transcriptomic splicing analysis

Reads were grouped by gene assignment for each sample, and the CIGAR sequences for their alignments were then used to generate a measure of splicing, wherein each position was defined by the number of spliced-in reads that included a base at the position (including mismatched bases) and the total number of reads that spanned across the position. Short regions in these CIGAR sequences (less than 10 bases) were removed by combining them with the preceding region, and insertions were ignored. To exclude a subset of reads that were found to be short, spurious alignments, reads that were more than 80% aligned to the reference were excluded. The first and last regions of each read were also ignored, which reduced the noise introduced by the variability in alignment of the 3′ and 5′ ends of reads. For each set of biological replicates, these splicing pileups were combined and grouped into distinct regions by calculating the cumulative average spliced-in fraction along the genomic index and starting a new region whenever the fraction differed from the cumulative average by more than 0.1. Additionally, genomic positions were excluded when the total read coverage fell below 5 for any individual sample or below 20 for the average coverage among biological replicates. These regions were then filtered to exclude regions that were not retained in any condition (regions with a mean splicing fraction less than 0.05 for all samples being analyzed). For each set of biological replicates, the number for total reads and spliced-in reads were both averaged across each region and rounded to integers. To estimate the retention rate for each region per experimental condition, a maximum likelihood estimate was calculated for the probability of being retained by minimizing the negative log likelihood function of the data on a negative binomial distribution. For each comparison between conditions, a maximum likelihood estimate was also calculated for the retention rate of the region for all samples between both conditions. To estimate per-region significances for each comparison, the probability of both samples having the same splicing rate was calculated by dividing the likelihood function of the negative binomial distribution fit to the data of both conditions by the product of the likelihood functions of the distributions fit to each condition separately.

### XBP1 splicing assay

RNA was collected as described above and then reverse-transcribed using SuperScript VILO Master Mix (Thermo Fisher Scientific #11755050). The resulting cDNA was diluted 1:10 and amplified by PCR with Taq polymerase (Thermo Fisher Scientific #10342020) using primers for the XBP1 intron region (see Table S1) using the following thermocycler program: 95 °C for 1 min, 34×[ 95 °C for 30s, 58 °C for 30s, 72 °C for 30s ]. PCR products were separated on a 3% agarose gel (half low-melt agarose) stained with SYBR Safe (Thermo Fisher Scientific S33102) and imaged on an Azure Biosystems 300 imager. The intensities of the bands for XBP1u, XBP1s, and their hybrid dimer product were each quantified and corrected against the local background gel intensity using ImageJ, and XBP1 splicing was calculated as (XBP1s + hybrid/2)/(XBP1u + XBP1s + hybrid). This same method was used for the MEF XBP1 splicing analysis, but with the mXBP1 primers instead and an annealing temperature of 60 °C.

### qPCR

RNA was collected as described above in biological triplicate and then reverse-transcribed using SuperScript VILO Master Mix (Thermo Fisher Scientific#11755050). The resulting cDNA was diluted 1:10 and used as template for qPCR using PowerUp SYBR Green Master Mix (Thermo Fisher Scientific A25741) and 500 nM primers (see Table S1). This mix was then run in duplicate on either an Applied Biosystems 7500 Fast RT-qPCR or an Azure Biosystems Cielo RT-qPCR with the following qPCR program: 95 °C for 5 min, 40×[ 95 °C for 30s, 58 °C for 30s, 72 °C for 30s, measure fluorescence ]. The resulting fluorescence data was then used to fit a mathematical model of PCR amplification^122^ in a custom python script to estimate the starting quantity of template in arbitrary units. This estimate was then averaged between the technical duplicates, normalized against the averaged estimate of beta-actin from the same cDNA, and divided by the actin-normalized value for the baseline sample. Lastly, the mean and sample standard error of this ratio was calculated across the biological replicates.

### Splicing PCR

RNA was collected as described above and then reverse-transcribed using SuperScript VILO Master Mix (Thermo Fisher Scientific#11755050). The resulting cDNA was diluted 1:10 and amplified by PCR using Phusion polymerase (New England Biolabs M0530S) with 500 nM primers flanking the alternatively spliced region of each gene (see table XX) using the following thermocycler program: 98 °C for 1 min, 35×[ 98 °C for 10s, 56 °C for 30s, 72 °C for 15s ], 72 °C for 2 min. PCR products were separated on a 3% agarose gel stained with SYBR Safe (Thermo Fisher Scientific S33102) and imaged on an Azure Biosystems 300 imager. The intensities of the bands for the spliced and unspliced products were each quantified and corrected against the local background gel intensity using ImageJ.

### Immunoblotting

Cells were plated in 6 cm dishes at 6e5 cells per sample and incubated for 24 h in supplemented DMEM, then for 24 h in supplemented Fluorobrite medium with 300 nM doxycycline, then given treatments of light or tunicamycin before being washed and scraped into cold PBS. These cells were then centrifuged and resuspended in 60 μl lysis buffer (50 mM Tris, 150 mM NaCl, 0.15% SDS, 1% Triton-X, 1 mM PMSF, and PhosStop phosphatase inhibitor (Roche)), incubated for 30 min on ice, and centrifuged at 2e4 RCF for 5 min. Protein in the supernatant was quantified by Pierce BCA assay (Thermo Fisher Scientific #A654530). Samples were incubated at 95 °C for 5 min with 5× loading dye (30% glycerol, 200 mM Tris pH 8.0, 10% SDS, 0.01% bromophenol blue, 40 mM DTT). For the normal PAGE, samples were loaded into 7.5% SDS-PAGE gel, separated by electrophoresis (120V, 120 min, 4 °C), and transferred to a nitrocellulose membrane *via* wet blotting (100V, 90 min, 4 °C). For the Phos-tag, lysate was loaded into 7.5% SDS-PAGE gel with 25 μM Phos-tag (NARD Institute AAL-107) and 50 μM MnCl_2_, separated by electrophoresis (100V, 180 min, 4 °C), and transferred to a nitrocellulose membrane *via* wet blotting (120V, 150 min, 4 °C). The membranes were blocked with 5% fat-free milk powder in TBST (20 mM Tris, 150 mM NaCl, 0.1% Tween 20, pH 7.6) for 1h at room temperature and then incubated with anti-IRE1 antibody in 5% milk/TBST overnight at 4 °C with agitation. The membranes were washed 3× with TBST, incubated with the secondary antibodies in 5% milk/TBST for 1h at room temperature, washed 3× with TBST, developed with SuperSignal West Femto (Thermo Fisher Scientific#34095), and imaged on an Azure Biosystems 300 imager for 5 min. Membranes were then stripped with 0.2 M NaOH for 3 min, washed several times with ddH_2_O, and blocked and probed as before with anti-Vinculin antibody.

## Data availability

The code used to process and analyze the raw transcriptomic and qPCR data and to generate the figures in this paper is freely available through Zenodo (https://doi.org/10.5281/zenodo.17781036), along with the source qPCR data, raw gel and blot images, and raw microscopy movies. Instructions for how to use the python scripts are in the included README.md file and detailed comments throughout the scripts and IPython notebooks. Design files for the cell illuminator are also available through Zenodo, in the same repository. Demultiplexed transcriptomic data and per-sample gene counts are deposited in the public National Center for Biotechnology Information Gene Expression Omnibus repository under the data identifier GSE302406. Raw nanopore data and the cell lines used in this paper are available upon request.

## Supporting information

This article contains supporting information.

## Conflicts of interest

The authors declare that they have no conflicts of interest with the contents of this article.

## Supporting information

Supplement final

Reagents and Materials
